# SVCT2 Promotes Neural Stem/Progenitor Cells Migration Through Activating CDC42 After Ischemic Stroke

**DOI:** 10.3389/fncel.2019.00429

**Published:** 2019-09-19

**Authors:** Yang Yang, Kaiyuan Zhang, Xuezhu Chen, Ju Wang, Xuejiao Lei, Jun Zhong, Jishu Xian, Yulian Quan, Yongling Lu, Qianying Huang, Jingyu Chen, Hongfei Ge, Hua Feng

**Affiliations:** ^1^Department of Neurosurgery and Key Laboratory of Neurotrauma, Southwest Hospital, Third Military Medical University (Army Military Medical University), Chongqing, China; ^2^Clinical Medical Research Center, Southwest Hospital, Third Military Medical University (Army Military Medical University), Chongqing, China

**Keywords:** ischemic stroke, neural stem/progenitors cells, ascorbic acid, sodium-vitamin C cotransporter 2, migration

## Abstract

Ischemic stroke is one of the most leading diseases causing death/long-term disability worldwide. Activating endogenous neural stem/progenitors cells (NSPCs), lining in the subventricular zone (SVZ) and dentate gyrus, facilitates injured brain tissue recovery in both short and long-term experimental settings. While, only a few proliferated NSPCs migrate toward the lesions to enhance endogenous repair after ischemia. Here, the results indicated that the functional recovery was evidently improved and the infarct volume was significantly reduced with ascorbic acid (AA) treatment in a dose-dependent manner from 125 to 500 mg/Kg, and the suitable therapeutic concentration was 250 mg/Kg. The possible mechanism might be due to activating sodium-vitamin C cotransporter 2 (SVCT2), which was down-regulated in SVZ after ischemia. Furthermore, immunostaining images depicted the number of migrated NSPCs from SVZ were significantly increased with 250 mg/Kg AA treatment or SVCT2 overexpression under the physiological and pathological condition *in vivo*. Besides, the data also represented that 250 mg/Kg AA or SVCT2 overexpression facilitated NSPCs migration *via* promoting F-actin assembling in the manner of up-regulating CDC42 expression using oxygen-glucose deprivation *in vitro*. Collectively, the present study indicates that SVCT2 promotes NSPCs migration through CDC42 activation to facilitate F-actin assembling, which enlarges the therapeutic scope of AA and the role of SVCT2 in NSPCs migration after brain injury.

## Introduction

Ischemic stroke is one of the most leading diseases causing death/long-term disability worldwide and current epidemiological data indicate that the burden of this illness is going to increase in the coming decades, especially in developing countries (Bernstock et al., 2017). Once ischemia occurs, it quickly triggers various pathological cascades, including (1) irreversible neuronal injury associated with an “ischemic cascade” (Wei et al., 2017), (2) the disruption of local energy balance-related to variations in oxygen/glucose concentrations and the depletion of cellular energy stores (Brorson et al., 1999), (3) concomitant release of neurotransmitters, inflammatory cytokines, chemokines, and reactive oxygen species (Wang et al., 2007; Alawieh et al., 2015; Li and Yang, 2016). Increasing evidence has demonstrated that activating endogenous neural stem/progenitors cells (NSPCs), lining in the subventricular zone (SVZ) and dentate gyrus (DG), facilitates injured brain tissue recovery in both short and long-term experimental settings (Green et al., 2018; Huang and Zhang, 2018). The reasons may due to (1) the proliferation, migration of NSPCs toward lesions and integration into the damaged neurovascular network (Wei et al., 2017), (2) enhancement of neurotrophic factors to support survival of epibiotic neural and vascular cells in injured regions (Yamashita and Abe, 2016), (3) suppression of local inflammation and excitotoxicity in the acute phase after injury (Stonesifer et al., 2017; Huang and Zhang, 2018). However, previous studies also indicate that this physiological repair response related to endogenous NSPCs is far from ideal, as patients continue to experience various levels of physical and cognitive morbidity post-ischemic injury (Gregoire et al., 2015; Uchida et al., 2017; Hao et al., 2018). Hence, it is worth developing approaches to activate endogenous NSPCs.

Ascorbic acid (AA)-also known as vitamin C, which is present in high concentrations in the cerebrospinal fluid (CSF) (Pastor et al., 2013), is an important mediator regulating NSPCs activation via its receptor. Previous researches have shown that AA plays a significant role in embryonic cerebral development and in directing NSPCs differentiation (Lee et al., 2000; Volpicelli et al., 2004). Furthermore, the study represents that AA enhances the generation of authentic midbrain-type dopamine neurons with improved survival and functions from ventral midbrain-derived NSPCs for cell-based therapy in Parkinson’s disease (Wulansari et al., 2017). Sodium-vitamin C cotransporter 2 (SVCT2), which is mainly expressed in the nervous system (CNS), is detected in NSPCs located in the inner and outer SVZ, suggesting the effect of AA on NSPCs might due to modulating SVCT2. Most recently, the study has indicated that SVCT2 is expressed in NSPCs in SVZ, where is the discrete niche originating endogenous NSPCs for cell replacement remedy after ischemia (Silva-Alvarez et al., 2017). In addition, research demonstrates SVCT2 enhances NSPCs differentiation into neurons via amplifying vitamin C uptake (Pastor et al., 2013; Oyarce et al., 2018). However, the report also represents that NSPCs obviously proliferate in the SVZ, but only a few proliferated NSPCs migrate toward the lesions after ischemia (Moskowitz et al., 2010), indicating that promoting NSPCs migration toward the lesions is an evident issue for activating endogenous repair after ischemia. Whether SVCT2 is implicated in facilitating NSPCs migration and its underlying mechanism needs to be elucidated.

In the present study, we hypothesized that SVCT2 potentiated NSPCs migration *via* promoting F-actin assembling. The results indicated that the functional recovery was evidently improved and the infarct volume was significantly reduced with AA treatment in a dose-dependent manner from 125 to 500 mg/Kg, and the suitable therapeutic concentration was 250 mg/Kg. The possible mechanism might be due to activating SVCT2, which was down-regulated in SVZ after ischemia. Furthermore, immunostaining images depicted the number of migrated NSPCs from SVZ to olfactory bulb (OB) along the rostral migratory stream (RMS) were significantly increased with 250 mg/Kg AA treatment or SVCT2 overexpression under physiological *in vivo*. Meanwhile, 250 mg/Kg AA treatment or SVCT2 overexpression facilitates NSPCs migration and integration into infarct core under a pathological condition *in vivo*. In addition, the data also represented that 250 mg/Kg AA or SVCT2 overexpression facilitated NSPCs migration *via* promoting F-actin assembling in the manner of up-regulating CDC42 expression using oxygen-glucose deprivation (OGD) *in vitro*.

## Materials and Methods

### Mouse Middle Cerebral Artery Occlusion/Reperfusion Model and Treatment

All animal procedures were approved by the University Committee on Use and Care of Animals, Third Military Medical University (Army Medical University) (No. SYXK 2012-0002). All experiments were performed according to the Chinese Animal Welfare Legislation for Protection of animals used for scientific purposes. The model was established using middle cerebral artery occlusion (MCAO) as previously described (Meng et al., 2018). Briefly, a total of 120 C57BL/6 mice (100 mice used for research and 20 mice died during experiment) were anesthetized with 2% isoflurane/air mixture (1–2 L/min). A 2.0-cm silicone-coated 8-0 nylon suture was gently inserted from the external carotid artery stump to the internal carotid artery, stopping at the opening of the middle cerebral artery. The ligation was maintained for 120 min before cerebral blood flow was restored. Body temperature was maintained at 37 ± 0.3°C by a feedback-controlled heating pad during surgery. Mice were maintained free access to food and water after surgery. Neurological deficits were graded after the mice recovered using a four-point neurological deficit severity scale as previously described (Meng et al., 2018). Mice with scores of 2 to 3 were used in the subsequent studies. Sham-operated mice underwent the same procedure without inserting the suture into the internal carotid artery. Thereafter, the mice were randomly assigned to different groups. Various concentration of AA (Sigma-Aldrich, St. Louis, MO, United States) was dissolved in normal saline and was intravenously injected *via* tail vein for 7 days (once a day, from days 1 to 7) after MCAO.

### 2, 3, 5-Triphenyltetrazolium Hydrochloride (TTC) Staining

2, 3, 5-Triphenyltetrazolium hydrochloride (TTC) staining was performed based on previous procedures (Xiong et al., 2004) on day 7 after MCAO. Generally, brains were rapidly removed after anesthetization, sectioned coronally at 1 mm intervals, and incubated in 2% TTC (Sigma-Aldrich, St. Louis, MO, United States) dye. Infarction area was calculated *via* subtracting the normal area stained with TTC in the ischemic hemisphere from the area of the non-ischemic hemisphere. Infarct volume was measured by summing infarction areas of all sections and multiplying by slice thickness. All experiments and analyses were performed by individuals blinded to treatment groups.

### Behavioral Tests

Rotarod test was performed as described by our previous work (Yang et al., 2018). The speed was set to increase gradually from 5 to 35 rpm, and the latency to fall (or cling to and spin with the rod for three full rotations) within 3 min was recorded for statistical analysis. Three trials for each mouse were performed separated by 10 min. A latency less than 60 s 1 day before implementing MCAO was set as an exclusion criterion for surgery.

Corner test was conducted to evaluate the neurological deficits as previously described (Zhao et al., 2017). Briefly, mice were allowed to walk into a 30-degree corner. When exited the corner, the mice could turn either to the left or the right, and this choice was recorded. Trials were repeated 10 times with 1-min interval, and the percentage of right turns was calculated.

Beam-walking was performed to assess the ability of the animal to remain upright and walk on a narrow beam as previously described (Yang et al., 2018). All mice were trained on the beam 1 day before MCAO, and only mice whose paws slipped down the horizontal surface of the beam (foot faults) fewer than 10 times per 50 steps were used for experiments. The number of contralateral forelimb and hindlimb foot faults within 50 steps were counted and analyzed, and mice that took fewer than 50 steps after MCAO were excluded. All experiments and analyses were performed by individuals blinded to treatment groups.

### Adeno-Associated Virus (AAV) and Lentiviral (LV) Transduction for SVCT2 Overexpression or SVCT2 Interference *in vivo* and *in vitro*

After anesthetization, three microliters of rAAV-Ef1a-pA, rAAV-Ef1a-SVCT2-pA, and rAAV-U6-shRNA(SVCT2)-Ef1a-pA (BrainVTA Inc., Wuhan, China) were intraventricularly administered into the lateral ventricles (0.33 μl/min) using specific coordinates (0.2 mm posterior to bregma, 2 mm ventral to the skull, and 1 mm lateral to the sagittal line) with a stereotaxic frame 14 days before MCAO surgery. Neurospheres (5000/ml) were pre-treated with moderate rLV-CMV-pA, rLV-Ef1a-SVCT2-pA and rLV-U6-shRNA(SVCT2)-pA (BrainVTA, Inc., Wuhan, China) for 48 h *in vitro*. Then, they were seeded on PLO pre-coated 24-wells cell culture cluster. SVCT2 sequence is provided in their 5′→3′ orientation: CGGCATGGAGTCCTACAAT. The efficiency of interfering and overexpression of LV and AAV was determined through evaluating SVCT2 expression using immunoblotting.

### Immunostaining

For immunostaining, neurospheres or 25 μm brain frozen sections were fixed with 4% paraformaldehyde in 0.01M phosphate-buffered saline (PBS, ∼pH 7.4) for 2 h at room temperature and blocked with normal goat serum or with 0.5% v/v Triton-X 100 (Sigma-Aldrich, St. Louis, MO, United States) in PBS. Samples were incubated in primary antibodies, SVCT2 (1:100, HPA059314, Sigma-Aldrich, St. Louis, MO, United States), Tubulin (sc-73242, Santa Cruz Biotechnology, Santa Cruz, CA, United States), BrdU (MAB4072, Millipore, Darmstadt, Germany), DCX (ab-18723, Abcam, Cambridge, United Kingdom), SOX2 (ab97959, Abcam, Cambridge, United Kingdom), NG2 (ab50009, Abcam, Cambridge, United Kingdom) and GFAP (ab53554, Abcam, Cambridge, United Kingdom) for 16–18 h at 4°C. After washing, they were incubated in relative fluorescence secondary antibodies for 2 h at room temperature. Cell nuclei were counterstained with 4′-6-Diamidino-2-phenylindole (DAPI, Sigma-Aldrich, St. Louis, MO, United States) for 10 min at room temperature. Then, coverslips were mounted onto glass slides and the images were captured by confocal microscope (Carl Zeiss, Weimar, Germany) and examined using Zen 2011 software (Carl Zeiss, Weimar, Germany).

For BrdU immunostaining, brain sections were incubated in 2 N HCl at 37°C for 30 min, sections were washed in 0.1M borate solution (pH 8.5) twice for 10 min, incubated in 3% H_2_O_2_ for 30 min, and blocked with 5% normal goat serum for 1 h at room temperature.

### Immunoblotting

Brain tissues or neurospheres were lysed in 200 μl ice-cold RIPA (Sigma-Aldrich, St. Louis, MO, United States) supplemented with protease inhibitor cocktail (Roche, Indianapolis, IN, United States). The cell lysate was harvested and centrifuged at 10,000 *g* at 4°C for 20 min. The protein concentration was determined by an enhanced BCA Protein Assay Kit (Beyotime, Beijing, China). Proteins were separated by 10% SDS-PAGE under reducing conditions and electro-blotted to polyvinylidene difluoride membranes (Roche, Indianapolis, IN, United States). Then, the membranes were blocked in TBST (0.5% Tween-20 in Tris-buffered saline) containing 5% (w/v) non-fat dry milk at room temperature for 2 h. Afterward, the membranes were incubated in primary antibodies, SVCT2 (ab229802, Abcam, Cambridge, United Kingdom) F-actin (ab205, Abcam, Cambridge, United Kingdom), CDC42 (2466, CST, Danvers, MA, United States), β-actin (TA346894, Zsgb-bio, Beijing, China) and tubulin (sc-73242, Santa Cruz Biotechnology, Santa Cruz, CA, United States) for 16–18 h at 4°C. After washing, the membrane was incubated with horseradish peroxidase (HRP)-conjugated secondary antibody, and all membranes were detected by ChemiDoc^TM^ XRS + imaging system (Bio-Rad, Berkeley, CA, United States) using the WesternBright ECL Kits (Advansta, Menlo Park, CA, United States). Densitometric measurement of each membrane was performed using Image Lab^TM^ software (Bio-Rad, Berkeley, CA, United States).

### Bromodeoxyuridine Injection

To examine migration, mice were received three intraperitoneal BrdU injections (50 mg/kg) per day for three consecutive days and were killed 4 days after the last injection. The co-labeled of BrdU^+^ and DCX^+^ cells lining in peri-infarct and infarct core on confocal projection images were obtained from 25-μm-thick coronal slices. Meanwhile, the co-labeled of BrdU^+^ and DCX^+^ cells in RMS under physiological status were measured using 25-μm-thick sagittal slices. Cell counts were performed in four slices per brain to calculate the relative percentages of cells found in the two areas.

### Primary NSPCs Culture

A total of 16 E14.5 C57BL/6 mice were employed to obtain primary NSPCs as previously described (Ge et al., 2015, 2016). Briefly, the cortices were washed twice in DMEM with 10% fetal bovine serum (FBS, vol/vol, Hyclone, Logan, UT, United States) after incubation in 0.25% trypsin-EDTA (Hyclone, Logan, UT, United States) at 37°C for 30 min. Then, the tissue samples were triturated using a fire-polished Pasteur pipette and passed through a 100-μm Nylon cell strainer (BD Falcon, San Jose, CA, United States) after they were washed twice with Dulbecco’s Modified Eagle’s Medium (Hyclone, Logan, UT, United States). Cell suspensions were cultured in DMEM/F12 medium supplemented with B27 (GIBCO, Grand Island, NY, United States), 20 ng/ml EGF (Peprotech, Rocky Hill, NJ, United States) and 20 ng/ml FGF-2 (Peprotech, Rocky Hill, NJ, United States) at 37°C under humidified 5% CO_2_ condition as recommended. For passaging cells, neurospheres were harvested by centrifugation (300 rpm), dissociated in StemPro Accutase Cell Dissociation Reagent (GIBCO, Grand Island, NY, United States) and grown in the medium described above. NSPCs, used for all experiments in the present study, were from passage 3 to 5.

### F-Actin Assembling Detection

The F-actin assembling was assessed as previously described (Ge et al., 2016), samples were incubated in Alexa Fluor 488 conjugated phalloidin reagents (Life Technologies, Waltham, MA, United States) at room temperature for 30 min. Images were visualized with a confocal microscope (Carl Zeiss, Weimar, Germany) and measured using Zen 2011 software (Carl Zeiss, Weimar, Germany).

### NSPCs Migration Assays

To evaluate the influence of AA or SVCT2 overexpression on NSPCs migration, the neurospheres (5000/ml) were seeded on 6-wells cell culture cluster with PO pre-coated in complete medium for 24 h. The migration distance index was calculated by average migration distance/neurosphere diameter, the migrated cells index was calculated by a total number of migrated cells/neurosphere’s diameter. CDC42 selective inhibitor ZCL278 (50 μM, A8300, APExBIO, Boston, MA, United States) was added into the culture for 1 h before testing. The images were captured with phase-contrast microscope (Olympus, IX71, Tokyo, Japan) every 2 h after seeded for 24 h. And the results were analyzed by Image-pro plus 6.0 software.

### Statistical Analysis

All values were expressed as mean ± SEM, and the statistical analyses were conducted using SPSS v19.0 (SPSS, Inc., Chicago, IL, United States). Comparisons were analyzed using two-tailed Student’s *t*-tests. Behavioral data collected at repeating time points were analyzed using two-way ANOVA, followed by Tukey’s *post hoc* test. Other data for western blot, infarct volume and immunostaining were analyzed using one-way ANOVA, followed by Tukey’s *post hoc* test. A *P* < 0.05 represents significant difference.

## Results

### Ascorbic Acid (AA) Promotes Functional Recovery and Reduces Infarct Volume

**FIGURE 1:**
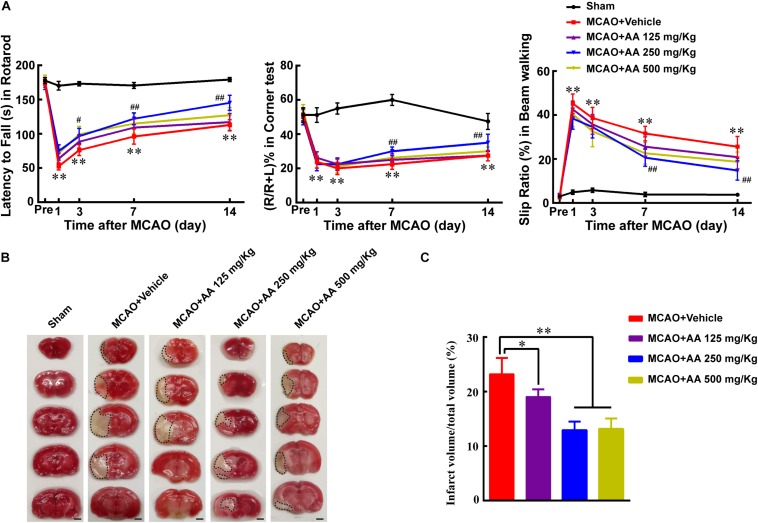
Ascorbic acid (AA) treatment promotes functional recovery *via* reducing infarct volume. **(A)** Quantitative data of behavioral tests: latency to fall (s) in rotarod (left), percentage of turn right (%) in corner test (middle) and the slip ratio (%) of the contralateral limbs within 50 steps in beam walking test (right) on days 1, 3, 7, and 14 in different groups: Sham, MCAO + Vehicle, MCAO + AA (125, 250, 500 mg/Kg) after ischemia, respectively. Data represented mean ± SEM, *n* = 4; ^∗^*P* < 0.05, ^∗∗^*P* < 0.01, significantly different from Sham; ^#^*P* < 0.05, ^##^*P* < 0.01, significantly different from MCAO + Vehicle. Two-way ANOVA followed by Tukey’s *post hoc* test. **(B)** Representative TTC staining images. White area with black dotted was infarct area. Scale bar: 2 mm. **(C)** Quantitative data of infract volume after ischemia in different groups: Sham, MCAO + Vehicle, MCAO + AA (125, 250, 500 mg/Kg). Data presented mean ± SEM, *n* = 3; ^∗^*P* < 0.05, ^∗∗^*P* < 0.01, significantly different from MCAO + Vehicle group. One-way ANOVA followed by Tukey’s *post hoc* test.

To investigate the effect of AA treatment on functional recovery after ischemia, three concentration of AA (125, 250, 500 mg/Kg) was used to certify the suitable therapeutic concentration. The results showed that group of moderate AA (250 mg/kg) significantly enhanced functional recovery using rotarod, corner and beam walking tests, compared with that in MCAO and AA (125 mg/kg) groups (Figure 1A). To understand the reason why AA treatment facilitates functional recovery, the infarct volume on day 7 was evaluated using TTC staining. The data indicated that a group of 250 mg/kg AA obviously decreased infarct volume, compared with that in MCAO and AA (125 mg/kg) groups (Figures 1B,C). Collectively, the results demonstrate that 250 mg/kg AA treatment potentiates functional recovery through reducing infarct volume. Hence, the dose of AA used in the present study was 250 mg/kg *in vivo* experiment.

### SVCT2 Down-Regulation Might Attenuate the Regenerative Ability Resulting From NSPCs After Ischemia

**FIGURE 2:**
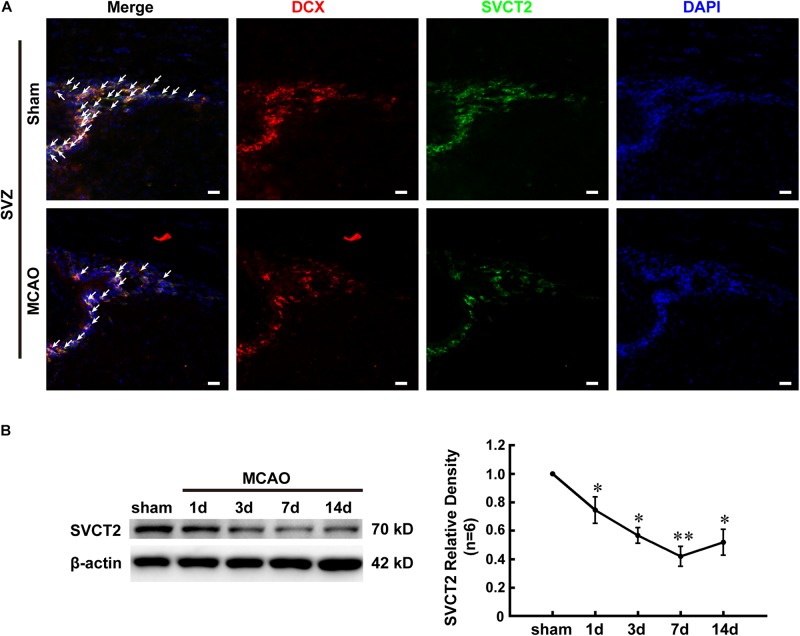
SVCT2 down-regulation might attenuate the regenerative ability resulting from NSPCs after ischemia. **(A)** Representative immunostaining of co-labeled DCX and SVCT2 at SVZ on day 7 in group Sham and MCAO. DAPI was used to label the nuclei. White arrows indicated co-labeled DCX^+^ and SVCT2^+^ NSPCs. Scale bar: 20 μm. **(B)** Representative immunoblotting images and quantitative analysis of SVCT2 at SVZ on days 1, 3, 7, and 14 in group Sham and MCAO. Data presented mean ± SEM, *n* = 3; ^∗^*P* < 0.05, ^∗∗^*P* < 0.01, significantly different from Sham. One-way ANOVA followed by Tukey’s *post hoc* test. SVZ, subventricular zone.

To understand why AA treatment improves the functional recovery and decreasing infarct volume, the endogenous NSPCs activation, which is a key spontaneous regenerative process that affects the infarct volume post-stroke, was evaluated. The SVCT2 expression in NSPCs was firstly determined by immunostaining on day 7. The results indicated that the number of co-labeled DCX^+^ and SVCT2^+^ NSPCs at SVZ on day 7 was significantly reduced in MCAO group than that in Sham group (Figure 2A). Meanwhile, immunoblotting bands depicted that SVCT2 expression was progressively reduced with time going on from days 1 to 14 and reached a nadir on day 7 after ischemia (Figure 2B).

### SVCT2 Over-Expression Benefits Functional Recovery *via* Facilitating NSPCs Migration

**FIGURE 3:**
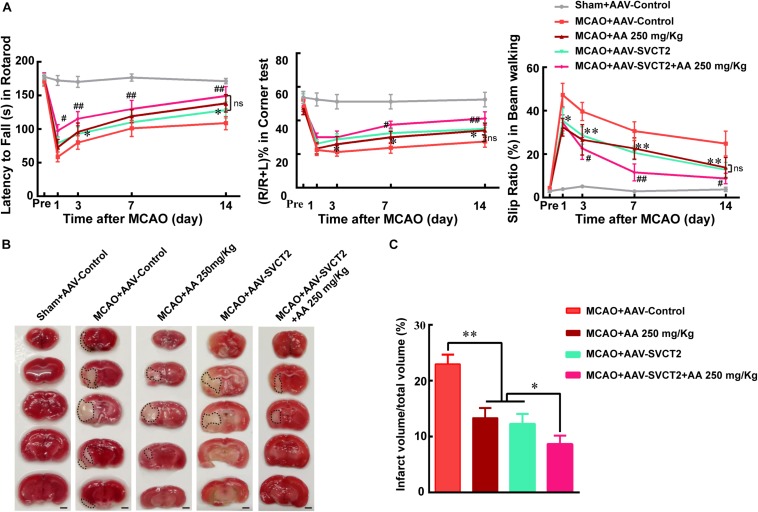
SVCT2 over-expression benefits functional recovery induced by AA treatment. **(A)** Quantitative data of behavioral tests: latency to fall (s) in rotarod (left), percentage of turn right (%) in corner teat (middle) and the slip ratio (%) of the contralateral limbs within 50 steps in beam walking test (right) on days 3, 7, and 14 after ischemia in different groups: Sham + AAV-Control, MCAO + AAV-Control, MCAO + 250 mg/Kg AA, MCAO + AAV-SVCT2, MCAO + AAV-SVCT2 + 250 mg/Kg AA. Data represented mean ± SEM, *n* = 4; ^∗^*P* < 0.05, ^∗∗^*P* < 0.01, MCAO + AAV-SVCT2 vs. MCAO + AAV-Control group; ^#^*P* < 0.05, ^##^*P* < 0.01, MCAO + AAV-SVCT2 + 250 mg/Kg AA vs. MCAO + 250 mg/Kg AA and MCAO + AAV-SVCT2. Two-way ANOVA followed by Tukey’s *post hoc* test. **(B)** Representative TTC staining images. White area with black dotted was infarct area. Scale bar: 2 mm. **(C)** Quantitative data of infract volume after ischemia in different groups: Sham + AAV-Control, MCAO + AAV-Control, MCAO + 250 mg/Kg AA, MCAO + AAV-SVCT2, MCAO + AAV-SVCT2 + 250 mg/Kg AA. Data represented mean ± SEM., *n* = 3; ^∗^*P* < 0.05, ^∗∗^*P* < 0.01. One-way ANOVA followed by Tukey’s *post hoc* test.

The above data implied that SVCT2 down-regulation impaired NSPCs migration from SVZ to deteriorate functional recovery. Hence, the SVCT2 up-regulation was used to certify the role of SVCT2 playing in NSPCs mobility. The results demonstrated that SVCT2 overexpression using AAV-SVCT2 (Supplementary Figure 1A) benefited functional recovery (Figure 3A) and decreased infarct volume on day 7 post-ischemia than that in MACO group (Figures 3B,C). Interestingly, the data also represented SVCT2 over-expression held the same beneficial effect as 250 mg/Kg AA treatment in decreasing the infarct volume and alleviating functional disorders (Figures 3A–C). Meanwhile, SVCT2 over-expression combined 250 mg/Kg AA treatment obtained the most advantages among all groups (Figures 3A–C).

**FIGURE 4:**
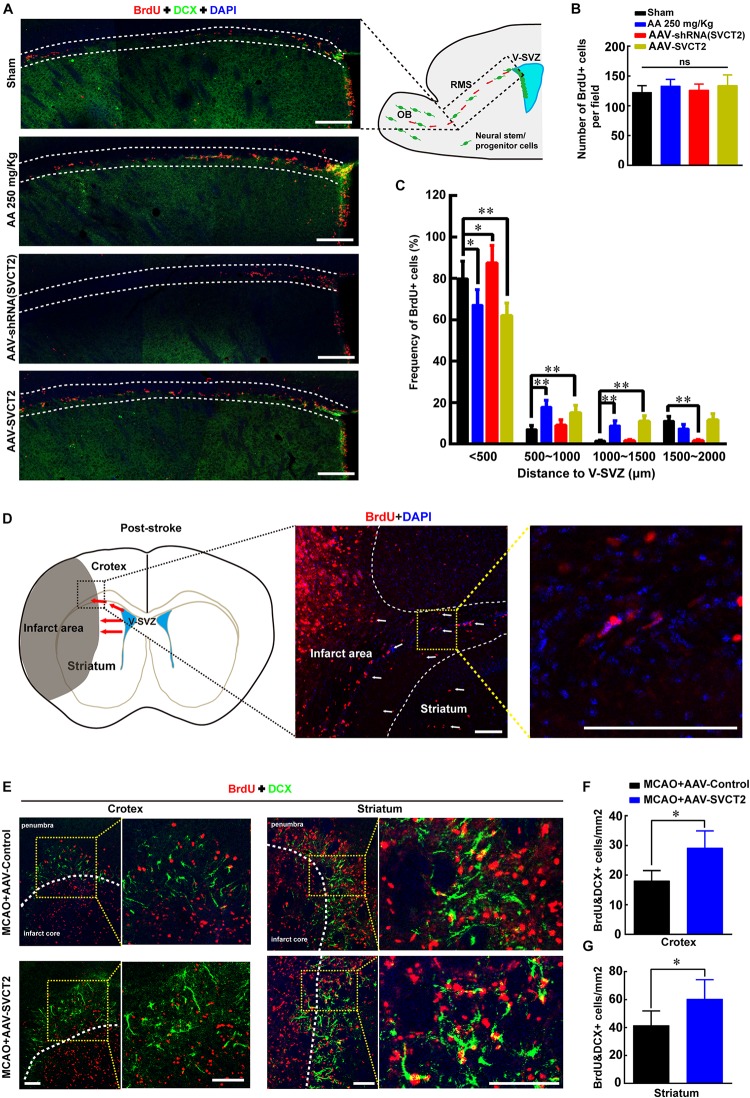
SVCT2 over-expression facilitates NSPCs migration *in vivo*. **(A)** Emblematic immunostaining of DCX^+^ and BrdU^+^ NSPCs along RMS (area between two white lines) without ischemia on day 7 in various groups: Sham, 250 mg/Kg AA, AAV-shRNA (SVCT2) or MCAO + LV-SVCT2. DAPI was used to label the nuclei. Scale bar: 200 μm. **(B)** Number of BrdU^+^ NSPCs along RMS per field in four groups. One-way ANOVA followed by Tukey’s *post hoc* test. ns, not significant. **(C)** Percentage of BrdU^+^ NSPCs along RMS at distances of 0–500, 500–1000, 1000–1500, and 1500–2000 μm away from V-SVZ. Data were expressed in mean ± SEM, *n* = 3; ^∗^*P* < 0.05, ^∗∗^*P* < 0.01, Two-way ANOVA followed by Tukey’s *post hoc* test. **(D)** Schematic image shows the migration route of NSPCs (red arrows) in coronal brain section after brain injury. **(E)** Representative immunostaining of DCX^+^ and BrdU^+^ NSPCs in cortex and striatum (yellow dotted grid indicates the observation area, and white dotted lines distinguish the infarct core and the penumbra) on day 7 after ischemia in group MCAO + AAV-Control and MCAO + AAV-SVCT2. Scale bar: 100 μm. **(F)** Quantitative analysis of BrdU^+^ and DCX^+^ cells around penumbra in cortex in group MCAO + AAV-Control and MCAO + AAV-SVCT2. Data were shown as mean ± SEM, *n* = 4; ^∗^*P* < 0.05, Student’s *t*-test. **(G)** Quantitative analysis of BrdU^+^ and DCX^+^ cells around penumbra in striatum in group MCAO + AAV-Control and MCAO + AAV-SVCT2. Data were shown as mean ± SEM, *n* = 4; ^∗^*P* < 0.05, Student’s *t*-tests. V-SVZ, ventricular-subventricular zone; RMS, rostral migratory stream; OB, olfactory bulb.

To unravel the underlying mechanism that AA treatment or SVCT2 over-expression decreases infarct volume to promote functional recovery, the effect of SVCT2 over-expression on NSPCs migration toward OB was firstly investigated using immunostaining under physiological condition. The data revealed that the number of BrdU^+^ cells was not significantly different among four groups (Figures 4A,B). While, the amount of migrated NSPCs from SVZ to OB along RMS was significantly increased in group AA treatment and SVCT2 over-expression, and the enhanced effect in SVCT2 over-expression group was equal to that in AA treatment group (Figures 4A,C). Meanwhile, down-regulation of SVCT2 using AAV-shRNA (Supplementary Figure 1A) evidently suppressed NSPCs migration, compared to Sham group (Figures 4A,C).

Next, the role of SVCT2 in regulating NSPCs migration under pathological condition was also elucidated using MCAO on day 7 post-ischemia using immunostaining. Herein, the number of BrdU^+^ and DCX^+^ NSPCs was evaluated between cortex and striatum at penumbra after ischemia as shown in schematic image (Figure 4D). The results illustrated that the number of co-labeled NSPCs was significantly increased in cortex and striatum at penumbra in MCAO + AAV-SVCT2 group than that in MCAO + AAV-Control group (Figures 4E–G). Collectively, SVCT2 plays an important role in NSPCs migration *in vivo*.

### SVCT2 Plays an Important Role in NSPCs Migration *in vitro*

To explore the role of SVCT2 in NSPCs migration *in vitro*, OGD experiment was performed to mimic ischemia. Firstly, the NSPCs were isolated from E14.5 mice, and its characteristics were identified according to our previously established protocol (Supplementary Figures 2A–D). Then, the cultured neurospheres were incubated in various concentrations to investigate the effect of AA on NSPCs migration for 24 h using phase-contrast microscope. The AA dose was as follows: 200, 400 μM and 1, 5 mM. The results indicated that 400 μM AA significantly promoted NSPCs migration in distance and cell number (Supplementary Figures 3A–C).

**FIGURE 5:**
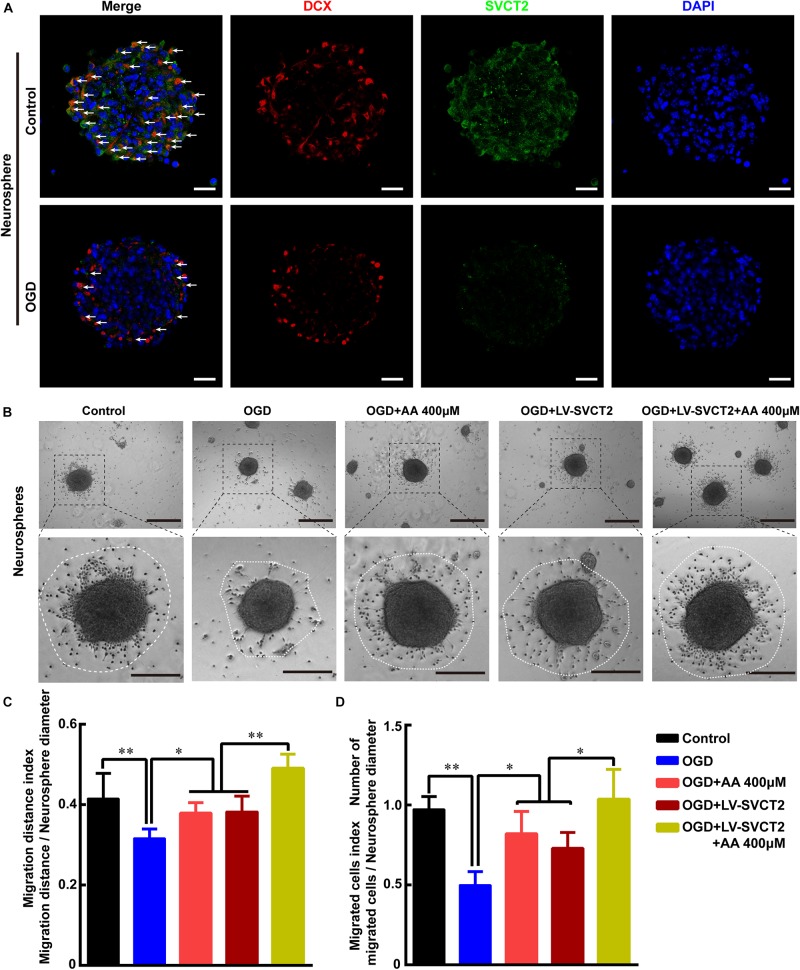
Oxygen-glucose deprivation (OGD) restrains NSPCs migration, while 400 μM AA or SVCT2 over-expression abrogates this inhibitory effect *in vitro*. **(A)** Representative immunostaining of DCX and SVCT2 in neurospheres after OGD treatment. White arrows indicated DCX^+^ and SVCT2^+^ cells. Scale bar: 50 μm. **(B)** Neurospheres were plated in PLO pre-coated 24-well plates with different treatments and captured by phase contrast microscopy after 24 h. Scale bar: 200 μm. Insets were magnified images from each photograph at high magnification. Scale bar: 100 μm. Quantitative analysis of NSPCs migration distance **(C)** and number of migrated cells **(D)**. Data represented mean ± SEM, *n* = 6; ^∗^*P* < 0.05, ^∗∗^*P* < 0.01. One-way ANOVA followed by Tukey’s *post hoc* test; ns, not significant.

Furthermore, the data showed that SVCT2 expression was evidently reduced in OGD group, compared to control group (Figure 5A). Meanwhile, the data revealed that the migrated cell number and migration distance was obviously reduced under OGD condition (Figures 5B–D) or SVCT2 knockdown by shRNA (Supplementary Figure 1B). While this inhibitory effect was greatly eliminated with 400 μM AA treatment or SVCT2 over-expression (Figures 5B–D and Supplementary Figures 4A–C) using LV-SVCT2 (Supplementary Figure 1B), especially in group of SVCT2 over-expression combined with 400 μM AA treatment (Figures 5B–D). Together, these data certify that the level of SVCT2 expression could greatly influence NSPCs migration.

### SVCT2 Furthers F-Actin Assembling to Potentiate NSPCs Migration *via* Up-Regulating CDC42 Expression

**FIGURE 6:**
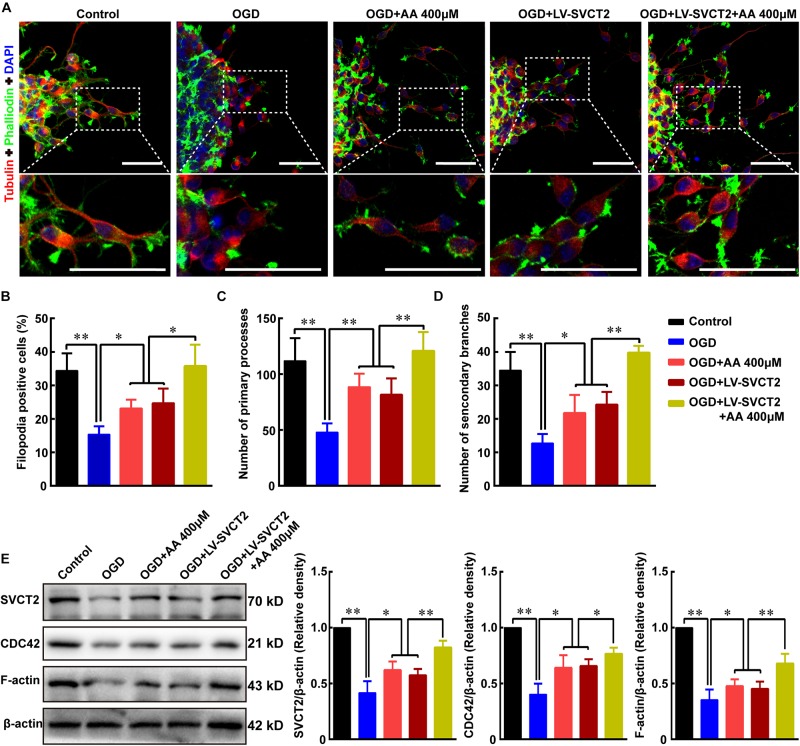
SVCT2 furthers F-actin assembling to potentiate NSPCs migration *via* up-regulating CDC42 expression. **(A)** Representative immunostaining of α-tubulin and phalloidin after neurospheres migration for 24 h in various groups: Control, OGD, OGD + 400 μm AA, OGD + LV-SVCT2, and OGD + LV-SVCT2 + 400 μm AA. Insets were magnified images from each photograph. Scale bar: 50 μm. **(B)** Bar graph summarized the percent of F-actin assembling after different treatments. Quantitative analysis of average number of primary leading processes **(C)** and second branches **(D)** after above treatments, respectively. **(E)** Bands showed expression of SVCT2, CDC42 and F-actin after above treatments and β-actin was served as an internal control. Data were shown as mean ± SEM, *n* = 6; ^∗^*P* < 0.05, ^∗∗^*P* < 0.01. One-way ANOVA followed by Tukey’s *post hoc* test; ns, not significant.

To further understand the potential mechanism under SVCT2 over-expression promotes NSPCs migration, the percentage of F-actin assembling was assessed using phalloidin staining. Meanwhile, Tubulin, which plays a significant role in modulating cell polarization and regulating cell movement (Ge et al., 2016), was also stained to evaluate the primary processes and secondary branches formation. The data showed that the percent of F-actin assembling, the number of primary processes and secondary branches were significantly reduced under OGD condition (Figures 6A–D). While this inhibitory effect could be abrogated with 400 μM AA treatment or SVCT2 over-expression, particularly in group of SVCT2 over-expression combined with 400 μM AA treatment (Figures 6A–D).

Next, the expression of SVCT2, CDC42 and F-actin was evaluated using immunoblotting assays. Bands illustrated that the expression of SVCT2, CDC42 and F-actin were decreased under OGD condition (Figure 6E). However, these proteins were up-regulated using 400 μM AA treatment, SVCT2 over-expression and combined use of SVCT2 over-expression and 400 μM AA (Figure 6E).

**FIGURE 7:**
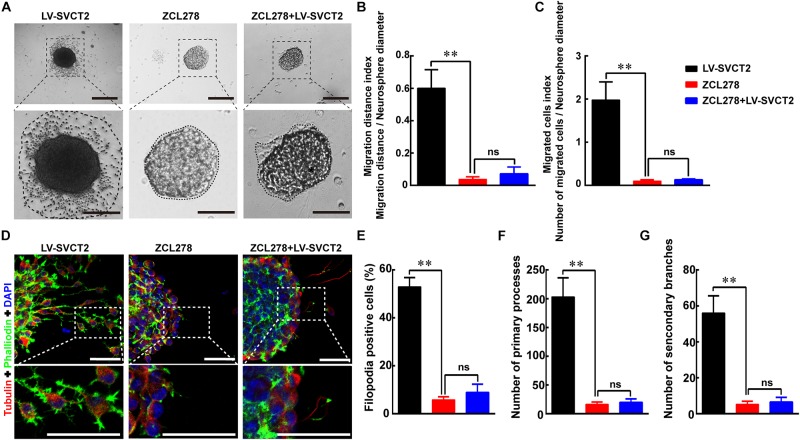
ZCL278 blocks NSPCs migration induced by SVCT2 overexpression. **(A)** Neurospheres migration after 24 h in different groups: LV-SVCT2, ZCL278, and ZCL278 + LV-SVCT2. Scale bar: 200 μm. Insets were magnified images from each photograph. Scale bar: 100 μm. **(B,C)** Quantitative analysis of NPSCs migration distance **(B)** and number of migrated cells **(C)**. **(D)** Representative immunostaining of α-tubulin and phalloidin after neurospheres migration for 24 h in the following groups: LV-SVCT2, ZCL278, and ZCL278 + LV-SVCT2. Scale bar: 50 μm. Insets were magnified images from each photograph. Quantitative data of the percent of F-actin assembling **(E)**, average number of primary leading processes **(F),** and second branches **(G)** after above treatments respectively. Data were shown as mean ± SEM, *n* = 6; ^∗∗^*P* < 0.01. One-way ANOVA followed by Tukey’s *post hoc* test; ns, not significant.

In addition, ZCL278, one CDC42 selective inhibitor, abrogated the enhanced migration effect induced by 400 μM AA and SVCT2. The results indicated that the migrated cell number and outgrowth distance were obviously reduced with addition of ZCL278, and SVCT2 over-expression could not alleviate this inhibitory effect (Figures 7A–C). Thereafter, the percentage of F-actin assembling, primary processes and secondary branches formation were also decreased with addition of ZCL278, even with SVCT2 over-expression (Figures 7D–G). Collectively, these data suggest that SVCT2 facilitates NSPCs migration through up-regulating CDC42 expression to enhance F-actin assembling.

## Discussion

The previous report has indicated that cortex development relies on projection neurons, which originate from progenitors located in the cortex, whereas neuroblasts are born in the ventral domains of the telencephalon and then migrate tangentially to reach the cortex (Azzarelli et al., 2014). Meanwhile, increasing studies have demonstrated that the concentration of AA reaches its highest concentrations during embryonic neurogenesis (Schaus, 1957; Oyarce et al., 2018), suggesting that AA is an important regulator to mediate cerebral cortex development due to neuroblasts migration. Recently, several researches have shown that AA promotes NSPCs differentiation into neurons via its receptor SVCT2 (Pastor et al., 2013; He et al., 2015; Oyarce et al., 2018), implying that SVCT2 might play an evident role in directing NSPCs migration induced by AA.

In the present study, our data indicated that infarct volume was reduced due to NSPCs migration with AA treatment or SVCT2 overexpression, thereafter improved the functional recovery post-ischemia. To our limited knowledge, this is the first study to identify the role of SVCT2 in potentiating endogenous NSPCs migration. Endogenous NSPCs are a neural subtype that holds the capacity of neuronal replacement (Wei et al., 2017), suppression of local inflammation (Stonesifer et al., 2017; Huang and Zhang, 2018) and enhancement of neurotrophic factors secretion after brain injury (Yamashita and Abe, 2016), suggesting that NSPCs is a promising therapeutic strategy post-stroke. However, previous study has shown that NSPCs obviously proliferate in the SVZ, but only a few proliferated NSPCs migrate toward the injured region after ischemia (Moskowitz et al., 2010). Hence, current research opens up a new horizon for the use of AA in rebuilding injured neurovascular network.

Ascorbic acid, serving as an antioxidant, plays a protective role against oxidative stress after stroke (May, 2012). Most recently, studies have indicated that AA supplementation nearly reduces the risk of cerebral vascular diseases (CVDs) (Chen et al., 2013; Al-Khudairy et al., 2017), implying that AA treatment is a premature treatment for stroke. Here, the results-SVCT2 overexpression with AA treatment significantly reduced infarct volume in the present research-possibly give an answer why AA is a premature treatment for stroke is that the low expression of SVCT2 post-ischemia, which strengthens uptake of AA, blocks the AA intake to protect neural cells loss after brain injury. Another reason for infarct volume reduction may due to the benefit of AA against oxidative stress after stroke, which has been certified by previous studies (Manzanero et al., 2013; Chamorro et al., 2016). Our data also indicated that SVCT2 overexpression with AA treatment significantly promoted NSPCs migration.

The mechanism underlying SVCT2 overexpression or AA treatment promotes NSPCs migration is mediating F-actin assembling through up-regulating CDC42 expression. The direct effect of F-actin assembling is to regulate cytoskeleton to facilitate cell mobility. Various factors affect F-actin assembling such as the Rho family of GTPases, which act as core regulators of cell migration via regulating intracellular actin dynamics (Warner et al., 2018). CDC42, one of three members of the Rho GTPases (CDC42, Rac1, and RhoA), is a key regulator of actin dynamics, functioning to connect multiple signals to actin polymerization (Ridley, 2016; Watson et al., 2017). Here, our data unravel that SVCT2 overexpression or AA treatment enhances F-actin assembling to regulate NSPCs migration through up-regulating CDC42 expression, which is consistent with previous studies (Watson et al., 2017; Warner et al., 2018). Moreover, ZCL278 is a small molecule that specifically targets CDC42–ITSN interaction and inhibits CDC42-mediated cellular mobility. Our results also indicated that the migrated cell number and outgrowth distance were obviously decreased with addition of CDC42 selective inhibitor ZCL278, even with AA treatment. However, the inhibitory effect was not completely eliminated using SVCT2 overexpression or AA treatment, suggesting that some other mediators regulate NSPCs migration induced by SVCT2 over-expression, such as Rac1 and/or RhoA. Hence, the additional network will be deciphered in our future work. Previous researches have indicated that AA overdose results in dizziness, faintness, fatigue, and headache (Levine et al., 1995). Our results might answer this question is that AA in high concentration (5 mM) inhibit NSPCs migration (Supplementary Figures 3A–C).

Some limits need to be elucidated in our future work. First, the effect of AA and/or SVCT2 on NSPCs differentiation needs to be demonstrated. Second, previous researches have also revealed that SVCT2 expressed in neuron (Mun et al., 2006), astrocytes (Salazar et al., 2018), glial tanycytes (Garcia Mde et al., 2005), microglia (Portugal et al., 2017), oligodendrocyte (Guo et al., 2018), pericytes (Parker et al., 2015), Schwann cells (Gess et al., 2010), ependymal cells (Mun et al., 2006). Hence, we also believe that SVCT2 bears different functions in different cells and different diseases, except for AA transport and promoting NSPCs migration. Thereafter, the role of SVCT2 on other cell lineage after ischemia needs to be illustrated. In addition, whether SVCT2 exert different effect among different species should be certified in our future work.

In short, the present study indicates that SVCT2 promotes NSPCs migration through CDC42 activation to facilitate F-actin assembling, which enlarges the therapeutic scope of AA and the role of SVCT2 in NSPCs migration after brain injury.

## Data Availability Statement

The raw data supporting the conclusions of this manuscript will be made available by the authors, without undue reservation, to any qualified researcher.

## Ethics Statement

All animal procedures were approved by the University Committee on Use and Care of Animals, Third Military Medical University (Army Medical University) (No. SYXK 2012-0002). All experiments were performed according to the Chinese Animal Welfare Legislation for Protection of animals used for scientific purposes.

## Author Contributions

YY performed most of the experiments with assistance from KZ, XC, JW, XL, JZ, JX, YQ, and YL. YY analyzed the results and produced the figures. KZ conducted the MCAO and statistical analysis. JZ, JX, and YQ performed the cell culture and treatments. XC, JW, QH, and XL performed the immunoblotting and immunostaining. YL conducted the ascorbic acid injection *via* tail vein. JC wrote the preliminary draft of the manuscript. YY and RH edited the manuscript. HG and HF designed the experiments and helped to writing the manuscript. All authors approved the final version of the manuscript.

## Conflict of Interest

The authors declare that the research was conducted in the absence of any commercial or financial relationships that could be construed as a potential conflict of interest.

## Supplementary Material

FIGURE S1 The efficiency of interfere and overexpression of SVCT2 using AAV or LV transfection. **(A)** Representative immunoblotting indicating down-regulation SVCT2 using AAV-shRNA (SVCT2) transfection or up-regulation by AAV-SVCT2 transfection *in vivo*. **(B)** Representative immunoblotting indicating down-regulation SVCT2 using LV-shRNA (SVCT2) transfection or up-regulation by pLV-SVCT2 transfection *in vitro*. Tubulin was served as an internal control. Data represented mean ± SEM, *n* = 3; ^∗^*P* < 0.05, ^∗∗^*P* < 0.01. One-way ANOVA followed by Tukey’s *post hoc* test.

FIGURE S2 Characteristics of NSPCs isolated from mice. **(A)** Cultured neurospheres were notably observed under phase contrast microscopy. Scale bar: 100 μm. **(B)** Representative immunostaining showed SVCT2 expressed in SOX2^+^ NSPCs. Scale bar: 50 μm. **(C)** Representative immunostaining showed neurospheres held the differentiation potential into NG2^+^ cells [oligodentrocyte progenitor cells (OPCs)]. Scale bar: 50 μm. **(D)** Representative immunostaining indicated cultured cells differentiated into DCX^+^ cells (Neurons) and GFAP^+^ cells (Astrocytes). Scale bar: 50 μm.

FIGURE S3 Ascorbic acid facilitates NSPCs migration in a dose-dependent manner. **(A)** Neurospheres were allowed to migrate in different AA concentration for 24 h and images were captured with a phase contrast microscopy. Scale bar: 200 μm. Insets were magnified images from each photograph. Scale bar: 100 μm. Quantitative analysis of NPSCs migration distance **(B)** and number of migrated cells **(C)**. Data were shown as mean ± SEM, *n* = 4; ^∗^*P* < 0.05, ^∗∗^*P* < 0.01. One-way ANOVA followed by Tukey’s *post hoc* test.

FIGURE S4 SVCT2 overexpression potentiates NSPCs migration induced by AA. **(A)** Representative NSPCs migration after 24 h in various groups: Control, LV-shRNA(SVCT2), 400 μM AA, LV-SVCT2, and LV-SVCT2 + 400 μM AA. Scale bar: 200 μm. Insets were magnified images from each photograph. Scale bar: 100 μm. Quantitative analysis of NPSCs migration distance **(B)** and number of migrated cells **(C)**. Data were shown as the mean ± SEM, *n* = 6; ^∗^*P* < 0.05, ^∗∗^*P* < 0.01. One-way ANOVA followed by Tukey’s *post hoc* test.
