# Supplementary figures and images for: SVCT2 Promotes Neural Stem/Progenitor Cells Migration Through Activating CDC42 After Ischemic Stroke
Source: Front Cell Neurosci. 2019 Sep 19;13:429. doi: 10.3389/fncel.2019.00429 (PMC6761321; doi:10.3389/fncel.2019.00429)

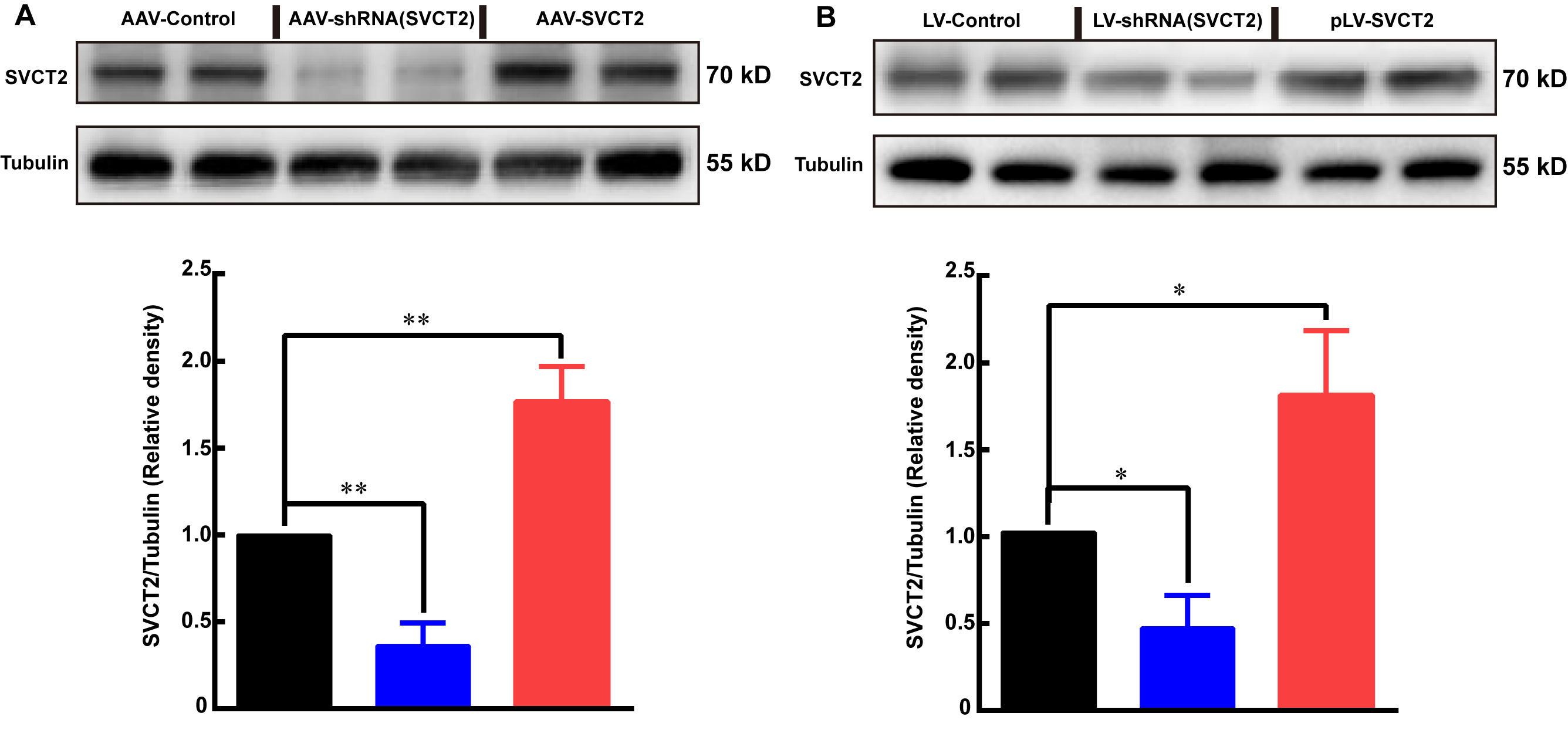

Supplement: FIGURE S1 — The efficiency of interfere and overexpression of SVCT2 using AAV or LV transfection. (A) Representative immunoblotting indicating down-regulation SVCT2 using AAV-shRNA (SVCT2) transfection or up-regulation by AAV-SVCT2 transfection in vivo. (B) Representative immunoblotting indicating down-regulation SVCT2 using LV-shRNA (SVCT2) transfection or up-regulation by pLV-SVCT2 transfection in vitro. Tubulin was served as an internal control. Data represented mean ± SEM, n = 3; ∗P < 0.05, ∗∗P < 0.01. One-way ANOVA followed by Tukey’s post hoc test. [file Image_1.JPEG]

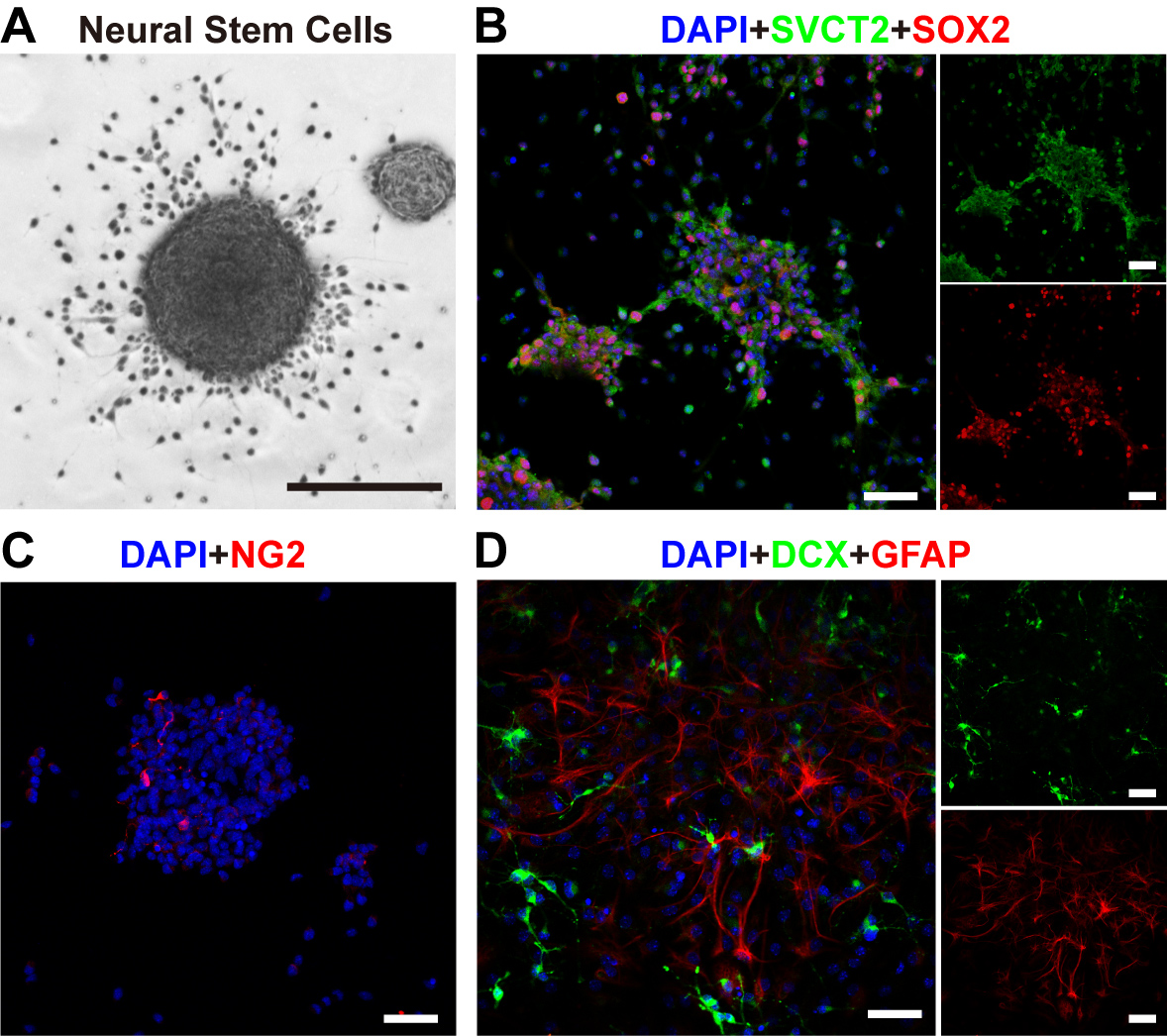

Supplement: FIGURE S2 — Characteristics of NSPCs isolated from mice. (A) Cultured neurospheres were notably observed under phase contrast microscopy. Scale bar: 100 μm. (B) Representative immunostaining showed SVCT2 expressed in SOX2+ NSPCs. Scale bar: 50 μm. (C) Representative immunostaining showed neurospheres held the differentiation potential into NG2+ cells [oligodentrocyte progenitor cells (OPCs)]. Scale bar: 50 μm. (D) Representative immunostaining indicated cultured cells differentiated into DCX+ cells (Neurons) and GFAP+ cells (Astrocytes). Scale bar: 50 μm. [file Image_2.JPEG]

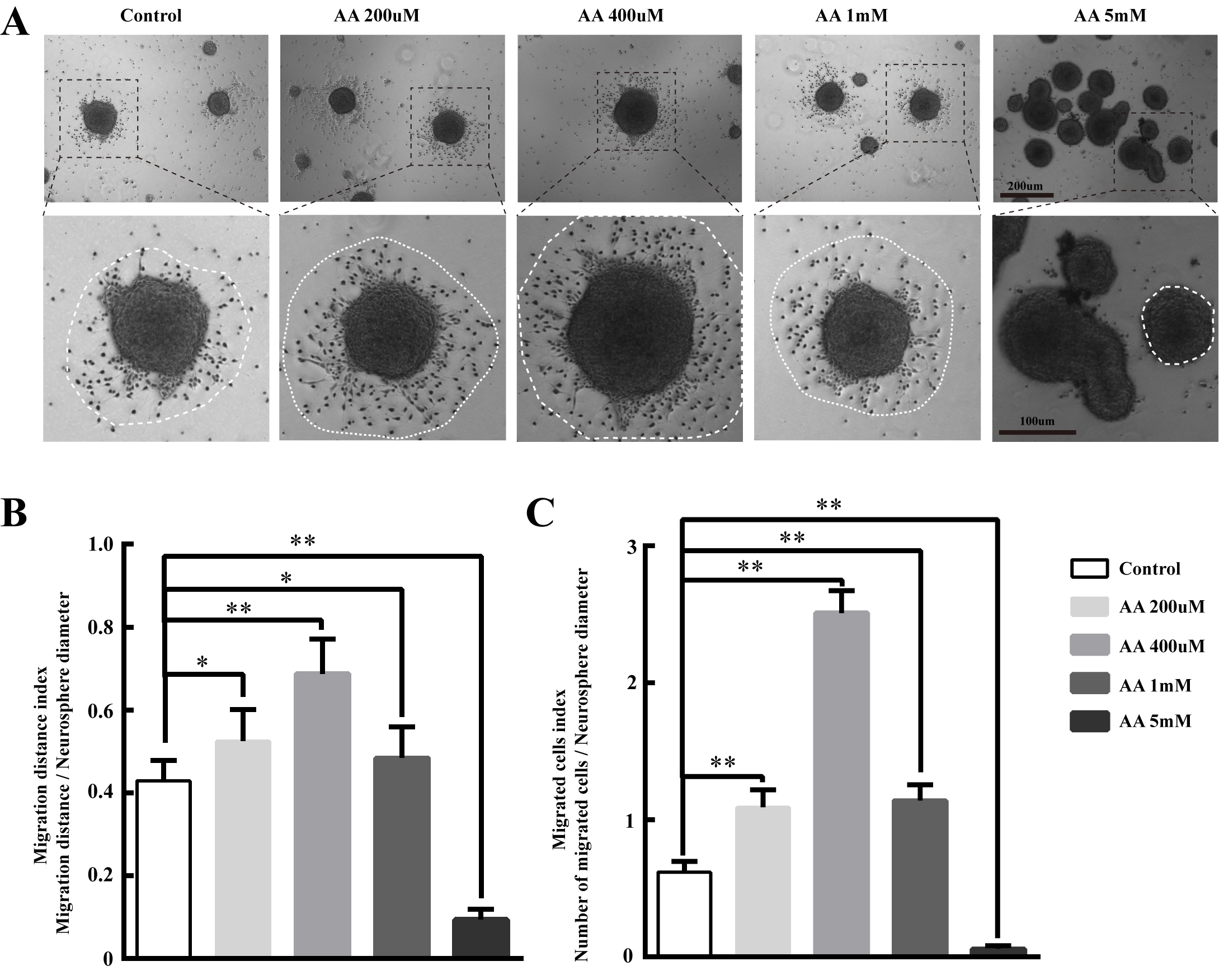

Supplement: FIGURE S3 — Ascorbic acid facilitates NSPCs migration in a dose-dependent manner. (A) Neurospheres were allowed to migrate in different AA concentration for 24 h and images were captured with a phase contrast microscopy. Scale bar: 200 μm. Insets were magnified images from each photograph. Scale bar: 100 μm. Quantitative analysis of NPSCs migration distance (B) and number of migrated cells (C). Data were shown as mean ± SEM, n = 4; ∗P < 0.05, ∗∗P < 0.01. One-way ANOVA followed by Tukey’s post hoc test. [file Image_3.JPEG]

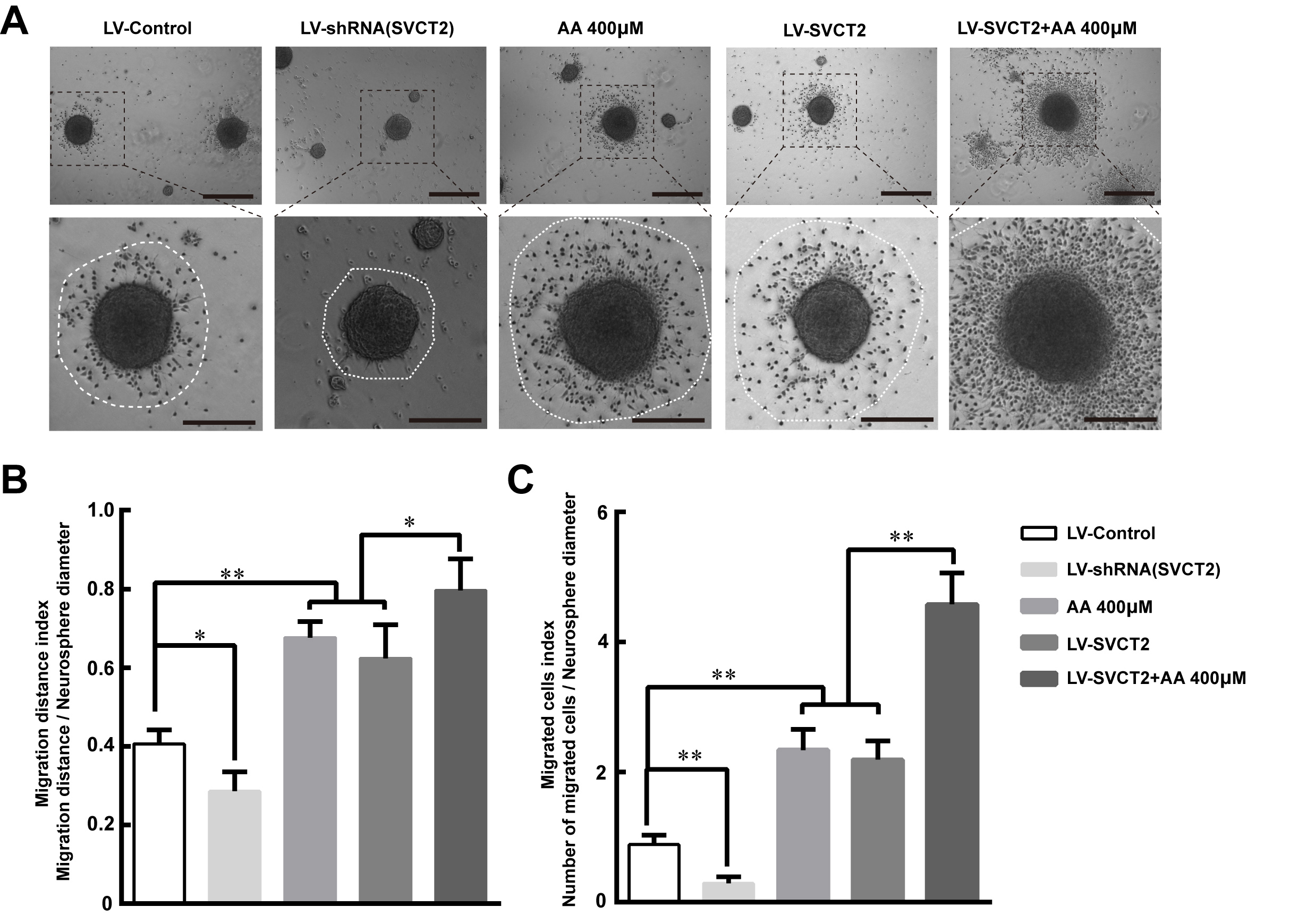

Supplement: FIGURE S4 — SVCT2 overexpression potentiates NSPCs migration induced by AA. (A) Representative NSPCs migration after 24 h in various groups: Control, LV-shRNA(SVCT2), 400 μM AA, LV-SVCT2, and LV-SVCT2 + 400 μM AA. Scale bar: 200 μm. Insets were magnified images from each photograph. Scale bar: 100 μm. Quantitative analysis of NPSCs migration distance (B) and number of migrated cells (C). Data were shown as the mean ± SEM, n = 6; ∗P < 0.05, ∗∗P < 0.01. One-way ANOVA followed by Tukey’s post hoc test. [file Image_4.JPEG]
